# Inpatient Specialist Palliative Care in Patients With Left Ventricular Assist Devices (LVAD): A Retrospective Case Series

**DOI:** 10.3389/fcvm.2022.879378

**Published:** 2022-06-29

**Authors:** Theresa Tenge, David Santer, Daniel Schlieper, Manuela Schallenburger, Jacqueline Schwartz, Stefan Meier, Payam Akhyari, Otmar Pfister, Silke Walter, Sandra Eckstein, Friedrich Eckstein, Martin Siegemund, Jan Gaertner, Martin Neukirchen

**Affiliations:** ^1^Department of Anesthesiology, Medical Faculty, University Hospital Duesseldorf, Heinrich Heine University, Duesseldorf, Germany; ^2^Interdisciplinary Centre for Palliative Medicine, Medical Faculty, University Hospital Duesseldorf, Heinrich Heine University, Duesseldorf, Germany; ^3^Department of Cardiac Surgery, University Hospital Basel, Basel, Switzerland; ^4^Department of Cardiovascular Surgery, Medical Faculty, University Hospital Duesseldorf, Heinrich Heine University, Duesseldorf, Germany; ^5^Department of Cardiology, University Hospital Basel, Basel, Switzerland; ^6^Department of Palliative Care, University Hospital Basel, Basel, Switzerland; ^7^Department of Practice Development Nursing, University Hospital Basel, Basel, Switzerland; ^8^Intensive Care Unit, University Hospital Basel, Basel, Switzerland; ^9^Department of Clinical Research, University of Basel, Basel, Switzerland; ^10^Faculty of Medicine, University of Basel, Basel, Switzerland; ^11^Palliative Care Center Hildegard, Basel, Switzerland

**Keywords:** heart assist devices, left ventricular assist devices, heart failure, palliative care, end of life care, quality of life, cardiac surgery

## Abstract

**Background:**

Repeat hospitalizations, complications, and psychosocial burdens are common in patients with left ventricular assist devices (LVAD). Specialist palliative care (sPC) involvement supports patients during decision-making until end-of-life. In the United States, guidelines recommend early specialist palliative care (esPC) involvement prior to implantation. Yet, data about sPC and esPC involvement in Europe are scarce.

**Materials and Methods:**

This is a retrospective descriptive study of deceased LVAD patients who had received sPC during their LVAD-related admissions to two university hospitals in Duesseldorf, Germany and Basel, Switzerland from 2010 to 2021. The main objectives were to assess: To which extent have LVAD patients received sPC, how early is sPC involved? What are the characteristics of those, how did sPC take place and what are key challenges in end-of-life care?

**Results:**

In total, 288 patients were implanted with a LVAD, including 31 who received sPC (11%). Twenty-two deceased LVAD patients (19 male) with sPC were included. Mean patient age at the time of implantation was 67 (range 49–79) years. Thirteen patients (59%) received LVAD as destination therapy, eight patients (36%) were implanted as bridge to transplantation (BTT), and one as an emergency LVAD after cardiogenic shock (5%). None of the eight BTT patients received a heart transplantation before dying. Most (*n* = 13) patients lived with their family and mean Eastern Cooperative Oncology Group (ECOG) performance status was three. Mean time between LVAD implantation and first sPC contact was 1.71 years, with a range of first sPC contact from 49 days prior to implantation to more than 6 years after. Two patients received esPC before implantation. In Duesseldorf, mean time between first sPC contact and in-hospital death was 10.2 (1–42) days. In Basel, patients died 16 (0.7–44) months after first sPC contact, only one died on the external sPC unit. Based on thorough examination of two case reports, we describe key challenges of sPC in LVAD patients including the necessity for sPC expertise, ethical and communicative issues as well as the available resources in this setting.

**Conclusion:**

Despite unequivocal recommendations for sPC in LVAD patients, the integration of sPC for these patients is yet not well established.

## Introduction

Heart failure remains one of the leading causes of death worldwide (1, 2). Despite optimized pharmacological treatment and heart transplantation (HTX), implantation of mechanical circulatory support (MCS) often presents the last therapeutic option (3). In 2011, 355 HTX were performed and 693 MCS were implanted in Germany, compared to 340 HTX and 843 MCS in 2020 (4). Left ventricular assist devices (LVAD) are the most commonly used MCS option (4). The implantation of a LVAD can be based on different intentions. In bridge to transplant (BTT) patients, the LVAD provides hemodynamic support until possible HTX. Destination therapy (DT) is intended for patients for whom HTX is not an option. Both concepts, BTT and DT, prolong survival and enhance the quality of life (5). However, patients experience enormous physical and psychosocial distress and often suffer from LVAD-related complications, such as bleeding, driveline infection, or pump thrombosis, which lead to re-hospitalization (5). Data from the Interagency Registry for Mechanically Assisted Circulatory Support (INTERMACS) report an overall LVAD 1-year mortality of 20% and a 2-year mortality of 70% (6). The dying process can be complicated and discussions about deactivation of a LVAD can be challenging and burdensome for patients, families, and health care teams (7). Knowledge about optimal end-of-life care in LVAD patients is scarce (7).

Palliative Care (PC) is defined by the World Health Organization (WHO) as “an approach that improves the quality of life of patients and their families facing the problems associated with life-threatening illness […]” (8). While all medical professions and disciplines provide general PC, specialist PC (sPC) is provided by a multi-professional sPC team (e.g., physicians and nurses with specialized education, psychologists and social workers) to in- and outpatients (9). Besides improving symptom control, the integration of sPC has been shown to be beneficial concerning shared decision making, defining and documenting end-of-life wishes (advanced care planning), and reducing distress of patients and relatives along their care pathway (9). Despite the fact that LVAD therapy is challenging for patients and their families (10), integration of sPC remains low (11) and European data on sPC involvement in the care for patients with LVAD are scarce (12, 13). Nevertheless, the European Society of Cardiology (ESC) generally recommends a palliative care consultation in all patients in the advanced stages of heart failure and for those considered for MCS or HTX before such interventions as a matter of protocol (3). The European Association of Palliative Care endorses in an expert position statement a needs assessment approach and to evaluate for sPC need during the regular heart failure visits (14). In the United States (U.S.), the issue of sPC in MCS was already addressed in 2010 by a clinical competence statement of a special task force (15). Mandatory sPC involvement in the DT-LVAD process is recommended since 2013 (16). The American Heart Association (AHA) guidelines also support the integration of sPC even *before* LVAD implantation (17). Since the publication of these recommendations in 2013, the involvement of sPC in the care of patients with LVAD has increased in BTT and DT (11, 18). A retrospective analysis in the U.S. from 2006 to 2014 showed an overall rate of 4% of LVAD patients received sPC and highlighted a significant increase of sPC involvement in 2014 to 7.2% (11). Nonetheless, to date there is no standardized sPC integration algorithm for LVAD patients (12, 19). Woodburn et al. developed a routine for sPC involvement in the DT-LVAD process before implantation and observed benefits for patients, caregivers, and clinicians (19). Therefore, sPC should not only be involved in end-of-life care, but also before the last year of life (20). Early integration of sPC (esPC) before LVAD implantation, in particular, could improve shared decision-making. However, availability of esPC is not yet widespread. Studies focusing on the need for sPC in BTT patients are rare (18). In addition to studies that evaluate these needs, the timing and format of sPC involvement in LVAD patients and investigations on the *status quo* in different countries are needed.

In our retrospective, descriptive study, we collected data from two university hospitals (Germany and Switzerland) to further explore the integration of sPC in deceased LVAD patients. Our main aims were to: (1) assess the extent to which LVAD patients received sPC, (2) determine how early an sPC is involved in the care trajectory, (3) identify the characteristics of patients who received sPC, (4) assess where sPC took place, and (5) identify the key challenges in end-of-life care.

## Materials and Methods

This study was performed after approval of the local ethics committee of the Medical Faculty of Heinrich-Heine-University Duesseldorf, Germany (Study-Nr.: 2021-1600). An additional ethical approval by the Ethics Committee of Northwestern- and Central Switzerland was not required (Req-2021-01368). We followed the Enhancing the QUAlity and Transparency Of health Research (EQUATOR) network guidelines for retrospective observational studies (here: STROBE) (21). All deceased adult patients who underwent BTT or DT LVAD implantation since 2010 were included. Also, the possible inclusion of sPC before LVAD implantation was studied. We defined integration of sPC before LVAD implantation as early sPC (esPC). Of the patients who had received sPC, only the subgroup of deceased patients was studied due to better comparability. Patients with other MCS, an already explanted LVAD and patients aged < 18 years were excluded. In Duesseldorf, no written informed consent was required for participation due to the retrospective and anonymized nature of this study, because patients already provide it with the treatment contract. In Basel, some LVAD patients refused general research consent and were therefore not included in our study. Medical records from the two institutions were electronically explored for the following information: Number of implants in total, number of LVAD patients receiving sPC and number of deaths this latter group of patients. Assessable patient characteristics included sex, age, Eastern Cooperative Oncology Group (ECOG), LVAD concept and LVAD device, date of the LVAD implantation, date and place of first sPC contact, and date and place of death. Data were organized and analyzed in Microsoft Excel 2020 (version 16.42, Microsoft Corp., Redmond, WA, United States) using descriptive statistics. Figures were created using MATLAB (2021b, MathWorks Inc., Natick, MA, United States).

### Specialist Palliative Care Setting

Duesseldorf and Basel have long established sPC services. sPC involvement consists of patients’ contact to sPC nurses, to an sPC physician, and if needed to other sPC team members (e.g., psychological and social support, physiotherapy, creative therapy, and/or spiritual care). Both sPC teams have regular multiprofessional discussions about each patient’s needs and treatment goals. Patient visits take place in-hospital as consultation services on cardiothoracic surgery wards, intermediate care units (IMC), or intensive care units (ICU). Both centers also offer out-patient services. Patients may also be transferred to an sPC unit. In Duesseldorf, the sPC unit is in-house. In Basel, it is external with services provided by the Palliative Care Center Hildegard, Basel. The University Hospital Basel shares a close cooperation with this nearby facility. Both Duesseldorf and Basel offer esPC before LVAD implantation, sPC in LVAD is initiated by an interdisciplinary consensus of the participating clinicians as well as the patient and his next of kin.

## Results

### Left Ventricular Assist Devices Implantations, Rate of Specialist Palliative Care Involvement and Deceased Patients

**FIGURE 1:**
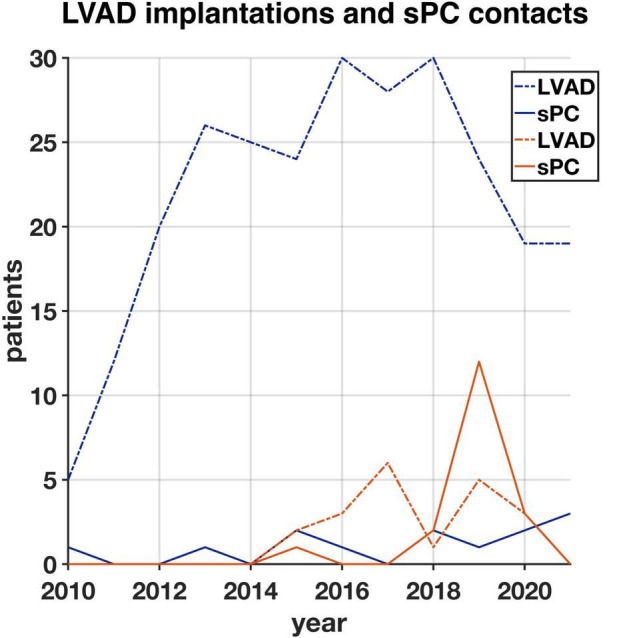
Number of all patients (with research agreement) who had a left ventricular assist device (LVAD) implanted from 2010 until 2021 in Duesseldorf (blue, *n* = 262) and Basel (orange, *n* = 20) and number of LVAD patients having their first specialist Palliative Care (sPC)-contact in each year in Duesseldorf (blue, *n* = 13) and Basel (orange, *n* = 18).

From 2010 to 2021, 262 patients underwent LVAD implantation at the University Hospital Duesseldorf, Germany and 26 at the University Hospital Basel, Switzerland. In Basel, 20 of these patients had provided a research agreement upon hospital admission. While 13 patients (5%) in Duesseldorf received sPC until December 2021, 18 patients (90%) in Basel received sPC during the same period. In Duesseldorf, 12 of 13 patients (92%) who had received sPC during their LVAD course had died. In Basel, ten of 18 (55%) of sPC patients died. Despite growing numbers of LVAD implantations in recent years (Figure 1), the proportion of LVAD patients receiving sPC in Duesseldorf remains low. In Basel, the cooperation between the LVAD and sPC teams has resulted in an increase of LVAD-sPC patients in 2018, but routine esPC was not realized in all patients. A total of 22 deceased LVAD patients who received sPC were included in the further analyses.

### Patient Characteristics

The deceased LVAD-sPC patients were primarily male (86%, *n* = 19), their mean age was 67 (range 49–79) years. In 73%, the diagnosis leading to LVAD implantation was ischemic cardiomyopathy (*n* = 16), whereas six patients (27%) suffered from dilated cardiomyopathy. At the time of first sPC contact, 13 patients lived with their family, four on their own, and two in nursing homes. Data about the place of living was not available for three patients. Mean ECOG performance status was three. In total, eleven patients (50%) had an ECOG status of four, marking a completely disabled patient who cannot carry on any selfcare and is confined to bed or chair. In Duesseldorf, none of the patients had an ECOG status of one or two, whereas in Basel 70% of the patients were able to carry out light work (ECOG 1) or selfcare (ECOG 2). In total, eight LVAD patients were originally considered for HTX (BTT). None of these patients were transplanted before death. Of the remaining 22 patients, 13 were implanted as DT (59%), and one was an emergency LVAD after cardiogenic shock (5%). Demographic and clinical characteristics are shown in Table 1.

**TABLE 1:** Patient characteristics (*n* = 22).

		All patients (*n* = 22)	Duesseldorf (*n* = 12)	Basel (*n* = 10)
Age (years), median (range)		67 (49–79)	65 (49–77)	69 (54–79)
Sex, *n* (%)	Male	19 (86.4)	11 (91.6)	8 (80)
	Female	3 (13.6)	1 (8.3)	2 (20)
LVAD concept,	BTT	8 (36.4)	7 (58.3)	1 (10)
*n* (%)	DT	13 (59.0)	4 (33.3)	9 (90)
	Emergency	1 (4.5)	1 (8.3)	0 (0)
LVAD device, *n* (%)	HeartWare^®^	16 (72.7)	7 (58.3)	9 (90)
	HeartMate III^®^	5 (22.7)	4 (33.3)	1 (10)
	HeartMate II^®^	1 (4.5)	1 (8.3)	0 (0)
ECOG, *n* (%)	0	0 (0)	0 (0)	0 (0)
	1	4 (18.1)	0 (0)	4 (40)
	2	3 (13.6)	0 (0)	3 (30)
	3	3 (13.6)	2 (16.6)	1 (10)
	4	11 (50)	9 (75)	2 (20)
	Missing	1 (4.5)	1 (8.3)	0 (0)
Place of living,	Alone	4 (18.1)	2 (16.6)	2 (20)
*n* (%)	Family	13 (59.0)	6 (50)	7 (70)
	Care home	2 (9)	1 (8.3)	1 (10)
	Other	3 (13.6)	3 (25)	0 (0)

*LVAD, left ventricular assist devices; BTT, bridge to transplant; DT, destination therapy; ECOG, Eastern Cooperative Oncology Group (performance status assessment score).*

### Specialist Palliative Care Involvement in Left Ventricular Assist Devices

**FIGURE 2:**
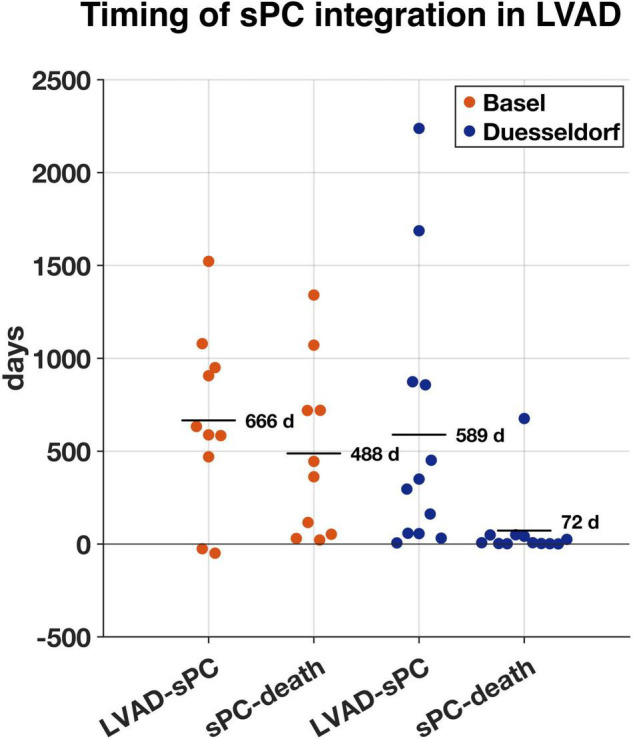
Time in days between left ventricular assist device (LVAD) implantation and first specialist Palliative Care (sPC)-contact (LVAD-sPC) and first sPC-contact until death (sPC-death) in Basel (orange, *n* = 10) and Duesseldorf (blue, *n* = 12). Horizontal lines represent mean duration in days.

**FIGURE 3:**
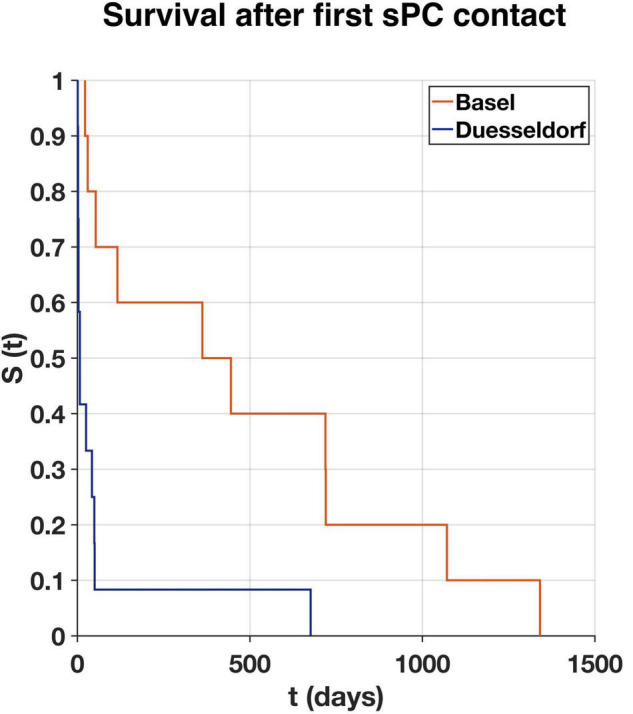
Survival rate (S) over time (t) of first specialist Palliative Care (sPC) contact to death in days as estimated according to the Kaplan-Meier method for Basel (orange, *n* = 10) and Duesseldorf (blue, *n* = 12). Median survival in Basel 404 days, in Duesseldorf seven days (log-rank test: *p* < 0.01).

Mean time between LVAD implantation and first sPC contact was 20.5 (−1.6 to 74.6) months (1.6 years in Duesseldorf and 1.8 years in Basel) (Figure 2). In Duesseldorf, all of the first sPC contacts took place on the IMC (*n* = 6) or ICU (*n* = 6), whereas in Basel the cardiothoracic surgery ward (*n* = 7) or the out-patient-clinic (*n* = 3) were places of first sPC contact. In Basel, two patients received esPC consultation 25- and 49-days prior to LVAD implantation, whereas in Duesseldorf esPC did not take place at all. In Duesseldorf, nine patients died in hospital, between one and 42 days after the first sPC contact (mean 10.2 days). All of these patients died of cardiovascular causes or LVAD complications (pump thrombosis: 2; infection of the driveline/LVAD: 2; bleeding: 2; cardiopulmonary failure: 2; hypoxic brain damage: 1). Three of these nine patients (33%) died on the ICU, five patients (55%) on the IMC, and one patient on a regular ward (11%). Three patients died out of hospital, the first patient at 49 days, the second (dependent on 24-h-intensive-care service at home) at 50 days, and the third 676 days after hospital discharge. In Basel, mean time between first sPC contact and death was 16 (0.7–44) months. The two esPC patients died 50 and 1,341 days after implantation. Five patients died on the cardiothoracic surgery ward, four on the ICU, and one patient on the external sPC unit (see “Case Report” below). In Duesseldorf, two patients were on the waiting list to be transferred to the sPC unit but died before admission. Survival time from first sPC contact is depicted in Figure 3 using the Kaplan-Meier method. Median survival in Basel and Duesseldorf was 404 and 7 days, respectively (log-rank test: *p* < 0.01).

The review of reasons or inquiries that led to sPC involvement as documented on the sPC requests revealed several aspects: discussion of goals of treatment, evaluating change to a palliative therapy approach, wish to die, burden and enhanced stress levels of caregivers and next of kin, early integration of sPC, and prolonged ICU stay.

Regarding the content and implementation of sPC consultations in LVAD patients, our data show sPC involvement in the following: assessment and management of symptom burden, psychological support of the patient and his or her next of kin, provision of spiritual care, support from social workers, exploring end-of-life wishes, advance care planning including advance directives and power of attorneys, and discharge planning. Data on the frequency and intensity of sPC contacts differ between study sites. In Duesseldorf, patients had sPC contact only during their index hospital stay and had a median time of direct sPC contact of 330 (270–1,500) min with incomplete data in three medical records. In Basel, patients had contact to the outpatient sPC clinic (*n* = 4) as well as during their inpatient stays. Data about the duration of each sPC contact are unavailable.

The following two case reports describe the course of two of the LVAD patients receiving sPC who died (Table 2). All identifying data have been removed and certain demographic data changed to protect privacy according to requirement of the local ethics committees.

**TABLE 2:** Patient characteristics of Case report 1 and 2.

	Case report 1	Case report 2
Age (years)	58	72
Sex	Male	Male
Diagnosis	ICM	DCM
LVAD-concepts	BTT	DT
Place of first sPC contact	IMC	Outpatient clinic
LVAD until sPC contact (years)	6.13	2.96
sPC contact until death (days)	3	362
Place of death	IMC	sPC unit
Context	Late sPC involvement in the LVAD process	LVAD deactivation

*ICM, ischemic cardiomyopathy; DCM, dilatative cardiomyopathy; LVAD, left ventricular assist devices; BTT, bridge to transplant; DT, destination therapy; sPC, specialist Palliative Care; IMC, Intermediate Care Unit.*

#### Case Report 1: Example of Late Integration of Specialist Palliative Care

A 58-year-old man with ischemic cardiomyopathy and multiple comorbidities had a HeartMate II^®^ LVAD implanted as a BTT 6 years ago. Before admittance to the hospital, the patient lived at home with his partner. Due to incurable Hodgkin-lymphoma, the patient was removed from the HTX waiting list 3 years ago. The patient was admitted to hospital because of LVAD thrombosis. He experienced new-onset headache due to a stroke. Replacement of the LVAD was discussed interdisciplinary. However, this option was abandoned after consideration of the patient’s life expectancy. During thrombolytic therapy, intracranial hemorrhage occurred, and the therapy had to be discontinued. After cessation of the thrombolytic therapy, LVAD-thrombosis progressed, and the patient developed sepsis. Given the situation, an interdisciplinary family conference involving the patient, his next of kin, cardiothoracic surgeons, and intensivists led to a decision to focus on comfort care and to integrate sPC. Consultation by the sPC team was requested by the treating physicians and a do not resuscitate/do not intubate (DNR/DNI) order was established. The sPC physician evaluated the patient to be imminently dying, assessed symptom control and spoke to the patient, the next of kin and the IMC clinicians. Together, they decided to initiate intravenous morphine via continuous application (100 mg morphine/50 ml 0.9% saline) with a rate of 2 mg/h due to persistent pain and stopped all other oral medication that did not provide symptom control. The patient was already used to opioids with a pain-treatment medication of ibuprofen, fentanyl 50 μg/h via a transdermal patch and requested his breakthrough pain medication fentanyl 100 μg buccal tablets three to four times a day. In case of fear or agitation, additional medication with midazolam was recommended by the sPC team. The patient was then pain free and fully orientated in communication. In the course of the following day the patient refused to eat and drank very little. After 3 days of increasing LVAD failure due to the progressing thrombosis and constant contact to the sPC physician and the sPC nursing team, the patient became more confused, but could still walk some steps and was not in pain. As he became agitated during the night due to reappeared pain and progressing dyspnea, intravenous morphine application was increased to 3 mg/h. After about an hour, the patient passed away next to his partner on IMC unit.

#### Case Report 2: End-of-Life Care and Deactivation of Left Ventricular Assist Devices on the External Specialist Palliative Care Unit

A 72-year-old man with ischemic cardiomyopathy, acute on chronic renal failure, and other comorbidities had a LVAD implanted as DT 4 years prior to admission. His first contact to the sPC outpatient-clinic was 3 years after implantation. Over time, the patient became severely impaired and led a bed-to-chair-existence due to weakness and dyspnea on exertion. He had been admitted to the university hospital due to deterioration of his general health condition weeks ago. During hospitalization, the patient suffered from hypoxia and *de facto* cardiac arrest due to unplanned disconnection of the LVAD batteries, performed by the patient himself. It remained unclear, whether this disconnection was performed consciously with suicidal intention or occurred accidentally. The patient was transferred to the ICU, regained cognition, ability to judge his situation, and was able to communicate. The sPC team was called for consultations. The patient reported physical distress due to breathlessness, fatigue, and severe anxiety due to worries about the future (“how can I go on like this”). Symptom control was established by the sPC team. In numerous round-table discussions over 2 weeks with the patient, his wife, and members from different disciplines, a shared decision-making process took place. At that time, the patient did not wish deactivation of the LVAD or escalation of other medical therapy, suicidal thoughts were denied. At that point, symptom-controlling measures were begun to promote wellbeing. The patient was then transferred to the external sPC unit. Before transferal, in collaboration with the cardio-technician who had been responsible for the patient for years, the sPC team was trained to manage and monitor the LVAD device.

In the sPC unit, the patient presented with anxiety, dyspnea, episodes of restlessness and delirium as well as neuropathic pain due to postherpetic neuralgia. Symptom control was established by the sPC team. Pain and dyspnea could be relieved with opioid therapy (hydromorphone 2 mg/day subcutaneous) in conjunction with non-opioids (dipyrone) and gabapentin. Also, the patient was treated with intravenous midazolam with a maximum of 8 mg/h during the night and 1 mg/h during the day due to anxiety and restlessness. The neurologic situation fluctuated, with intermittent phases of restlessness and disorientation. When the patient was awake and oriented, he often expressed a strong wish to die. His overall and neurologic situation declined further. After a multiprofessional meeting with the consulting ethicist, the wife, and the cardiology team, the decision to deactivate the LVAD under increased doses of opioids and benzodiazepines was made. The sPC physician inactivated the LVAD after being instructed by the perfusionist with intravenous propofol in standby for fast and deep sedation, whether this would have been necessary. The patient died within half an hour without signs of dyspnea, anxiety or other distress with his wife and the PC physician at his side.

Overall, the team of the sPC unit faced several challenges. The nursing team needed training and information about how to deal with the LVAD technically and about characteristics of the dying process of patients with an LVAD. Due to the unavailability of cardio-technicians or cardiology support during out-of-office hours in the sPC unit (not located on the university hospital campus), anxieties of the sPC nurses concerning the care of a patient with a LVAD device had to be addressed proactively and could be relieved.

## Discussion

In this study, we aimed to portray the current situation of sPC integration in LVAD patients in two German-speaking university hospitals. Notable findings of our investigation are as follows: (1) Consistent with the literature from the U.S. (11), we found that utilization of sPC in patients with LVAD generally remains low, with a strong discrepancy between centers. (2) Our data support the involvement of sPC in both DT and BTT patients. For DT patient, U.S. guidelines already recommend sPC prior to implantation. However, BTT patients can also benefit from sPC, for example when they experience a change of their treatment goal (HTX no longer intended) or even earlier. (3) The presented case reports show possible benefits of comprehensive and early sPC involvement, however, they also report challenges.

A retrospective analysis in the U.S. from 2006 to 2014 showed a 4% overall rate of sPC involvement in LVAD patients (11). Since the implementation of the U.S. guideline recommendation for sPC in DT-LVAD patients, this rate has significantly increased (11). In their retrospective study including 89 patients, Nakagawa et al. showed a significant increase with around 80% of BTT and DT patients receiving sPC in the last month of life (18). Although PC consultation prior to MCS implantation or HTX is suggested in the European guidelines in general, in a position manuscript published by the ESC, the situations “before LVAD implantation or transplant referral” are described as possible trigger for sPC, not as a mandatory recommendation as it is in the U.S. (22). In our study, sPC involvement occurred late in the LVAD process. Especially in Duesseldorf, it mostly occurred shortly before death. In Germany, the guideline for the treatment of chronic heart failure recommends an early and proactive screening of PC needs in patients with heart failure by the family doctor (23). Several potential reasons for the underutilization of sPC in heart failure have been identified. Many family doctors and cardiologists report lacking time for these conversations during primary care (24). Often, these clinicians refuse to talk to patients about their poor prognosis, and barriers exist among doctors to use the word “palliative” when talking to a patient (24). Also, the unpredictable disease trajectory of heart failure can promote a rather reactive use of sPC, most often during the latest phase of life (24). Crimmings et al. describe this current situation in the treatment of heart failure as a “death-denying culture” (24). Currently, a prospective, controlled multicenter study to explore the efficacy and cost-effectiveness of interdisciplinary sPC in symptomatic heart failure is in progress (25). Our study showed that despite the increasing trend of LVAD implantations at the two university hospitals, the integration of sPC in the care for LVAD patients is yet not well established. This discrepancy is highlighted by the 31 patients (11%) cared for with integration of sPC support among the 288 total LVAD implantations. Moreover, the disparity between the two centers, with only 5% of LVAD patients in Duesseldorf receiving sPC and 90% sPC involvement in Basel is revealing. Greater sPC involvement in Basel (especially after 2018) contributes to center-specific differences that make it difficult to compare the two cohorts and might partially explain a significantly longer survival after sPC involvement in Basel (Figure 3). Also, LVAD expertise in Basel is rather new (since 2014) and was established when there was already an awareness of sPC need in LVAD patients in the literature and international guidelines. Therefore, a closer collaboration between the LVAD and sPC teams exists, especially since 2018. In Duesseldorf, sPC is only involved when cardiac surgeons contact the sPC teams or when patients are being discussed on the weekly sPC team visit on the ICU. This present study might increase the awareness of members of the heart-teams to include sPC at an early stage in the LVAD trajectory.

Numerous positive predictors for sPC integration like DNR status, female sex, and metastatic cancer have been identified (11). Men receive LVAD three times more often than women (26). In this study, most patients were men, which reflects the existing gender gap in LVAD treatment (26). It may be that women have a clearer idea about their end-of-life wishes and are more likely to refuse LVAD therapy. Women might be more afraid of the burden for their caregivers and prefer to have a quiet and peaceful end-of-life period without an alarming LVAD device. Comorbidities such as metastatic cancer, psychiatric diseases or other serious comorbidities have not been investigated in this present study. However, these might influence the need of sPC in LVAD patients as they also affect the prognosis. Until admission to the hospital that resulted in sPC involvement, most patients (77%) lived with their family or alone. Their mean ECOG was three, which indicates that patients were only capable of limited selfcare. In Basel, where sPC involvement is more prevalent, 70% of patients had an ECOG status of one or two, indicating a fully active or mildly restricted patient. A proactive and earlier sPC involvement before LVAD implantation or the onset of complications leading to hospital admission may support advance care planning when patients still live in their familiar surroundings. The definition of trigger criteria for sPC might help to increase the rate of sPC integration in LVAD according to each patient’s needs.

Interestingly, most publications and the U.S. guidelines only focus on sPC in DT-LVAD patients (16, 19, 27). But in fact, the end-of-life issues of BTT und DT patients do not seem to differ significantly in terms of place of death, DNR orders, hospice enrollment, and PC during the last month of life (18). Our study shows that BTT patients might also have a need for sPC, since all eight presented LVAD BTT patients died without receiving HTX. When BTT patients experience a change of treatment goal (as presented in “Case Report 1” section), sPC can provide support especially during this phase.

Overall, our study shows late sPC involvement in the LVAD process. In Duesseldorf, the average time between first sPC contact and in-hospital death was around 10 days. During this rather short period, establishing a trusting relationship between the sPC team and the patient and their next of kin may be difficult. The AHA guidelines recommend starting sPC before implantation. In the context of cancer, early integration of palliative care has been shown to significantly prolong life and improve quality of life in a landmark study (28), while recent meta-analyses of cancer and non-cancer populations show no negative impact of esPC on survival (29, 30). Despite the cooperation between LVAD and sPC teams in Basel, only two patients had already received esPC before LVAD implantation, but in this center, the average time between first sPC contact and death was rather long compared to Duesseldorf. More data are needed to analyze the impact of esPC on the circumstances of death. Most patients (81%) died in hospital, mainly on the ICU or IMC ward, with just one dying on an sPC unit and three dying at home after hospital discharge. This finding may illustrate the fact that even despite esPC involvement, it is difficult to enable patients and their next of kin to die in their preferred place of death, which is known to be at home for most patients (31). Yet, earlier studies have found that significantly fewer LVAD patients die on the ICU after sPC involvement (18, 32).

The two case reports presented here demonstrate possible end-of-life scenarios as well as characteristics and challenges of sPC involvement. Concerning case report 1, the question occurs, why the patient was not presented to sPC earlier (e.g., when BTT changed to DT due to the non-curable comorbidity or even earlier, since BTT and DT patients experience similar distress and symptoms) (18). When LVAD thrombosis occurred in Case Report 1, the therapeutic focus still lay on life-sustaining intensive care therapy. Only after thrombolysis failed and sepsis occurred, end-of-life care and sPC integration was considered. As mentioned earlier, this sPC concept is rather reactive and suggests little anticipation of possible end-of-life scenarios and lack of screening for the patients’ PC needs. Therefore, esPC could help here to reduce such barriers. A standardized esPC concept in DT offers multiple benefits such as increased quality of life of patients, more advanced care planning, and enhanced satisfaction among clinicians (19). Case report 2 demonstrates that earlier involvement of the sPC team helped with shared decision making, supported the relatives, and facilitated establishment of a further integrated care pathway to allow treatment of the patient and his family concerning to their needs and wishes on a sPC unit. It was possible to show that management of the patient, his symptoms and his family may be performed on a continued pathway on a sPC unit. Yet, a necessary prerequisite for this involved intensive teaching of the external sPC team and close collaboration with cardio-technicians and cardiologists, which may not be available outside the cardiology center. Besides the required technical expertise, also ethical issues may arise during LVAD care, especially at end-of-life. On the one hand, an ongoing LVAD as a life sustaining technology might prolong natural dying and sPC team members might explore moral distress which is also observed in intensive care clinicians who care for MCS patients (33). On the other hand, LVAD deactivation may present an emotional situation for next of kin and all team members. An interdisciplinary and multiprofessional checklist that outlines different steps required for LVAD deactivation might help in these situations (34). In both centers, the sPC teams work as an interdisciplinary team. Thus, experts from different medical disciplines are included, e.g., from anesthesiology, psychosomatic medicine and oncology. The teams are multiprofessional and comprise doctors, nurses, psychologists, physical therapists, social workers, clerics and volunteers. Also included are other therapists for music therapy or animal assisted therapy. The team meets daily for a morning conference and monthly for supervision. Good communication between the team members and good integration of the different professions are considered crucial for a sustainable team. Each first patient contact starts with an assessment of symptoms by a sPC physician and a nurse, the patient’s history regarding the medical and personal background and the patient’s wishes and goals. Further patient contacts depend on the patient’s and the next of kin needs, which includes psychosocial support as well as physiotherapy or spiritual care. With routine implementation of sPC in an LVAD program, ideally there should be a 24/7 on-call support from the primary treating heart-team.

### Study Limitations

This is the first retrospective and descriptive study about sPC in LVAD patients in the German-speaking area. Limitations of this study are a limited number of patients as well as the fact that included patients were not compared to those who did not receive sPC. Therefore, we cannot observe an impact of sPC or esPC on, for example, the place or circumstances of death.

Another major limitation of our study is a possible selection bias resulting from the method of clinical database research. Only patients that ultimately received sPC and died in the process were included. Unfortunately, no information could be gathered about patients for whom sPC might have been discussed but ultimately was not provided. Also, data was collected from just two university hospitals in German-speaking countries and not from elsewhere in Europe. More data from other hospitals is needed to get a clear overview of the sPC situation in LVAD patients. In addition, the routine documentation process by each hospital’s sPC team is different. Therefore, a standardized and comparable assessment could not be provided. Both centers routinely use the ECOG to assess a patient’s performance status. This parameter was originally established in cancer patients and is not commonly used in heart failure patients. An analysis using parameters specific for heart failure patients seems more reasonable. However, cardiology-specific scores as the New York Heart Association (NYHA)-Classification have been shown to poorly discriminate between clinically important functional performance states in people with advanced heart failure (35). Additionally, other established physical performance tests in heart failure, such as the Six Minute Walking Test and the Timed Up and Go Test (36) focus primarily on functional activity rather than everyday-life performance as the ECOG does. The use of the Kansas City Cardiomyopathy Questionnaire (KCCQ) could also help in further studies to evaluate the health status (37). However, none of these parameters inform about uncovered needs that could require the involvement of sPC and therefore do not present suitable triggers to initiate sPC. The European Association for Palliative Care recommends sPC needs assessments in regular heart failure visits and advises to examine for “distressing symptoms, existential distress, recurrent heart failure exacerbation and progressive frailty or caregiver concerns” (14). Future studies are needed to identify specific triggers for standardized sPC in LVAD patients. Most obviously, as this is a retrospective real-world study, a control group is lacking. Although, case numbers are low and only two centers were involved, the observation of relatively large differences (e.g., in the time of sPC involvement) make the comparison between the two centers an interesting first step to study sPC involvement of LVAD patients. Due to the lack of generalizability with only two centers, certainly these differences have to be seen as site - rather than country - specific. To date, few sPC teams and very few sPC units or hospices are able to care for patients with LVAD. Therefore, this real-world pilot data may provide useful information for institutions planning to establish an sPC program for LVAD patients.

## Conclusion

In conclusion, although the rates of LVAD implantations have been growing in the last decade, the integration of sPC in the care for these patients is yet not well established. An increased awareness of the sPC need of LVAD patients has led to a proactive use of (e)sPC in one center. In general, sPC involvement still occurs relatively late in the LVAD process but has great potential for both BTT and DT patients. Our findings suggest that there remains a lack of sPC provision in LVAD patients in the German-speaking area, and further involvement of sPC should be pursued in the future.

## Data Availability Statement

The original contributions presented in this study are included in the article/supplementary material, further inquiries can be directed to the corresponding author.

## Ethics Statement

This study involving human participants was reviewed and approved by Ethics Committee of the Medical Faculty of Heinrich-Heine-University Duesseldorf, Germany (Study-Nr.: 2021-1600). An additional ethical approval by the Ethics Committee of Northwestern- and Central Switzerland was not required (Req-2021-01368). Written informed consent for participation was not required for this study in accordance with the retrospective nature of this study.

## Author Contributions

TT: concept and design, data collection, data analysis and interpretation, statistics, writing of the manuscript, and software. DSa: writing of the manuscript, data analysis and interpretation, and critical revision of the manuscript. DSc, MSc, JS, and SM: concept and design, supervision, and critical revision of the manuscript. PA: data collection, critical revision of the manuscript, supervision, and resources. OP: critical revision of the manuscript, supervision, and resources. SW: data collection, data analysis and interpretation, software, and critical revision of the manuscript. SE: concept and design, supervision, critical revision of the manuscript, and data analysis and interpretation. FE and MSi: critical revision of the manuscript, supervision, and funding. JG and MN: concept and design, writing of the manuscript, critical revision of the manuscript, and supervision. All authors contributed to the article and approved the submitted version.

## Conflict of Interest

DSa received speaker honoraria and educational grants from Abott and Medtronic as well as speaker honoraria from Abiomed and Nycomed. PA received speaker honoraria from Medtronic, Abbott, Edwards, Cryolife/Jotec, and Abiomed and has received research grants from Abbott and Edwards outside the submitted work. The remaining authors declare that the research was conducted in the absence of any commercial or financial relationships that could be construed as a potential conflict of interest.

## Publisher’s Note

All claims expressed in this article are solely those of the authors and do not necessarily represent those of their affiliated organizations, or those of the publisher, the editors and the reviewers. Any product that may be evaluated in this article, or claim that may be made by its manufacturer, is not guaranteed or endorsed by the publisher.

## Abbreviations

LVAD: left ventricular assist devices
BTT: bridge to transplant
DT: destination therapy
MCS: mechanical circulatory support
HTX: heart transplantation
sPC: specialist palliative care
esPC: early specialist palliative care
ECOG: Eastern Cooperative Oncology Group
ESC: European Society of Cardiology
INTERMACS: Interagency Registry for Mechanically Assisted Circulatory Support
AHA: American Heart Association
WHO: World Health Organization
DNR/DNI: do not resuscitate/do not intubate
IMC/ICU: intermediate care/intensive care unit.
